# Management of Synkinesis and Asymmetry in Facial Nerve Palsy: A Review Article

**Published:** 2014-10

**Authors:** Abbas Ali Pourmomeny, Sahar Asadi

**Affiliations:** 1 *Department of Physiotherapy, School of Rehabilitation Sciences, Isfahan University of Medical Sciences, Isfahan, Iran.*

**Keywords:** Bell's palsy, Electromyography biofeedback, Facial palsy, Physiotherapy, Synkinesis

## Abstract

**Introduction:**

The important sequelae of facial nerve palsy are synkinesis, asymmetry, hypertension and contracture; all of which have psychosocial effects on patients. Synkinesis due to mal regeneration causes involuntary movements during a voluntary movement. Previous studies have advocated treatment using physiotherapy modalities alone or with exercise therapy, but no consensus exists on the optimal approach. Thus, this review summarizes clinical controlled studies in the management of synkinesis and asymmetry in facial nerve palsy.

**Materials and Methods:**

Case-controlled clinical studies of patients at the acute stage of injury were selected for this review article. Data were obtained from English-language databases from 1980 until mid-2013.

**Results:**

Among 124 articles initially captured, six randomized controlled trials involving 269 patients were identified with appropriate inclusion criteria. The results of all these studies emphasized the benefit of exercise therapy. Four studies considered electromyogram (EMG) biofeedback to be effective through neuromuscular re-education.

**Conclusion:**

Synkinesis and inconsistency of facial muscles could be treated with educational exercise therapy. EMG biofeedback is a suitable tool for this exercise therapy.

## Introduction

The incidence of peripheral facial palsy is 23–35 cases in 100,000 (1). Half of all facial palsy cases are idiopathic (Bell´s palsy) and the remainder are caused by tumor, trauma, injury during surgery, herpes zoster virus, otitis, or Ramsay Hunt syndrome (2,3). Approximately 70% of cases of Bell’s palsy exhibit complete recovery within 3 months (4). Unfortunately, patients with incomplete recovery of facial nerve palsy (FNP) suffer from facial muscle weakness, contracture, hyperkinesis, atrophy, and synkinesis (1,5-7). Among these sequelae, facial synkinesis and asymmetry are the most common and the most serious both psychologically and socially (8), affecting the quality of life of the individual. 

Synkinesis is an abnormal involuntary associated facial movement (9-11) that occurs in nearly all cases of facial nerve degeneration which would tend to regenerate from the proximal site of injury (12). Synkinesis begins 3–4 months after regeneration of FNP and continues for up to 2 years (8-10). It is reported that 9–55% of patients exhibit incomplete recovery of FNP (9). Common types of facial synkinesis are oral-ocular synkinesis, involuntary eye closure during voluntary mouth movement and ocular-oral synkinesis, and involuntary mouth movement during voluntary eye closure (10). The etiology of synkinesis is not fully understood; however, aberrant regeneration of the facial nerve has been the most commonly reported cause (7,11,13).

Surgery (neurolysis and myoctomy), botulinum toxin-A (BTX-A) injection, and rehabilitation have been suggested for management of synkinesis (8). Neurolysis may relieve synkinesis temporarily, but the condition recurs, sometimes more severely than before (14). Myectomy, on the other hand, has a low recurrence rate, but has post-operative complications such as swelling, hematoma, lymphoedema, and ecchymosis (10). Furthermore, rehabilitation is always necessary after surgery.

Researchers have also suggested physiotherapy modalities, including electrical stimulation, massage, ultrasound, laser, and diathermy, with or without exercise therapy for FNP. In one of our previous studies, we investigated these modalities and highlighted the controversy in literature in which no form of treatment seemed to have priority (15). Therefore, a systematic review of FNP without spontaneous recovery or with incomplete recovery is necessary. The aim of this study is to investigate and review previous clinical studies (both case-control and randomized) in the management of synkinesis and asymmetry.

## Materials and Methods

English-language articles published up to 2013 and including the terms Bell’s palsy, FNP, synkinesis, rehabilitation, physic- otherapy, or botulinum toxin (BTX) in their title, abstract or keywords were extracted from databases including PubMed, Cinahl, Medline, EBMR, Google Scholar, Science Direct, and ProQuest. Among these articles, randomized case-controlled studies which investigated the sequelae of FNP and in which one of the treatment protocols was physiotherapy, rehabilitation, or neuromus- cular re-education were selected. Exclusion criteria included immediate introduction of treatment that precluded spontaneous recovery of Bell’s palsy. 

## Results

Following a comprehensive database search, 124 clinical articles concerning the management of FNP and synkinesis were identified. Cases of Bell’s palsy with spontaneous recovery were omitted. All studies except six were excluded because they did not match the inclusion criteria (Table. 1).

Among these six articles, four compared two different treatment protocols and the other two compared two similar treatment protocols. At least one of each group received neuromuscular re-education. Four studies used electromyogram (EMG) biofeedback. Seven groups received biofeedback. The treatment protocols were approximately similar and most of them used biofeedback; however the pathology, time of treatment, and evaluation method differed. Therefore, a meta-analysis was not possible, and it was necessary for greater convergence for a systematic review. Two hundred and sixty-nine (269) patients were selected. Group 1 (146 patients) received neuromuscular exercise and EMG biofeedback, while Group 2 (123 patients) acted as a control group and received mirror biofeedback or no treatment. None of the studies rejected exercise therapy and all of them demonstrated that it is effective in terms of facial symmetry; they also emphasized the value of EMG biofeedback.

**Table 1:** Characteristics of included studies

**Author and year**	**Methods of evaluation**	**Type of injury**	**Interventions**	**Result**
Beurskens 2006	Sunnybrook and House-Brackmann scale	Facial palsy	25 mime therapy, 25 without treatment	Mime therapy effective
Nakamura 2003	Recording and compare with sound side	Facial palsy	12 mirror biofeedback,15 without treatment	Mirror biofeedback effective
Pourmomeny 2013	Sunnybrook and House-Brackmann scale	Facial palsy	16 EMG biofeedback, 13 physiotherapy	EMG biofeedback effective
Toffola 2012	Sunnybrook scale	Facial palsy	38 EMG biofeedback, 35 mirror biofeedback	Both of them effective without significant difference
Ross 1991	Linear measurement of facial movement	Bell’s palsy	18 EMG biofeedback, 7 mirror biofeedback	EMG biofeedback effective
Toffola 2005	Sunnybrook and House-Brackmann scale	Bell’s palsy	28 stretch + active movement 37 EMG biofeedback	EMG biofeedback effective

*Beurskens* *in 2006* studied 50 patients who had been suffering from facial palsy, divided into two groups. The aim of this study was to investigate symmetry and synkinesis. Twenty-five patients received mime therapy, consisting of a mime and physiotherapy (thermotherapy and massage) which combined stimulation of facial emotional expression and functional movements. The aim of this therapy is to promote symmetry of the face at rest and during movement, enabling the patient to control synkineses or mass movements. The basics of this treatment is neuromuscular re-education training. Four main exercises (forehead wrinkle, smile, snarl, and lip pucker) with variations amplitudes and speeds were employed; exercise to one side of the face to control each movement, relaxation in the lower jaw, and exercise for mouth and eye with simultaneous inhibition of synkinesis (slow, small movement ) were included. A mirror was used for feedback. 

Mime therapy was performed for approximately 3 months, and 25 control patients received no treatment. All patients were evaluated using the House-Brackmann and Sunnybrook scales. The study showed a 20% improvement with the Sunnybrook scale and 6% improvement with the House-Brackmann in Group 1 (2).

*Nakamura in 2003* studied 27 patients in two groups. The aim of this study was to control and reduce synkinesis. Group 1 (12 patients) were instructed in mirror biofeedback. For example, during voluntary oral movement (lip pucker, smiling) involuntary eye movement should be prevented. The treatment period was 10 months (30 minutes daily exercise). Group 2 (15 patients) acted as controls and received no treatment. All patients were evaluated before and after treatment by recording and comparing the injured side with the sound side through measuring the percentage change on both sides of the face and the height of the interpalpebral space. The results showed that this educational protocol was successful in preventing synkinesis. FNP has several sequelae, but in this study the authors emphasize one type of synkinesis only and reported no data concerning facial symmetry (10).

*Pourmomeny in 2013* published a randomized trial in 29 patients who were divided into two groups after an electrodiagnosis test. The aim of this article was to investigate the symmetry and prevention of synkinesis. Sixteen patients in Group 1 were treated with EMG biofeedback for 1 year (daily for 1 month and once per week for 11 months). In the first step, EMG biofeedback was used to treat weak muscles (performed by attaching electrodes to the muscles with minimal tonicity and muscle strengthening performed using one channel). In the second step, when symptoms of synkinesis appeared, this modality was used to prevent synkinesis in addition to the treatment of weak muscles (using two channels, one for voluntary movement and the other for involuntary movement; the patient was asked to try to control synkinesis during active movement). The control group (13 patients) was permitted to undertake physical therapy in any rehabilitation center. Both groups were recommended home exercise therapy. Evaluation was performed using the Sunnybrook scale. The study showed improvement in both groups, but Group 1 showed significant differences compared with Group 2 in symmetry and reduction in synkinesis (16). 

*Dalla Toffola* *in 2012* investigated patients using electrodiagnosis. Cases of FNP with axonotmesis were included and those with neuropraxia were excluded. Seventy-three cases were selected. The aim of the study was to compare two types of exercise therapy. The patients divided into two groups. Group 1 (35 patients) were treated with mirror biofeedback, neuromuscular re-education, and exercise for prevention of synkinesis. Group 2 (38 patients) were treated with EMG biofeedback for strengthening the movement of the injured side and prevention of synkinesis. The study showed that both protocols were effective and caused improvement in symmetry and reduction in synkinesis with no significant differences between groups (17).

*Ross in 1991*conducted a study to compare EMG and mirror biofeedback. Twenty-five patients with residual Bell’s palsy at least 18 months after injury were selected. Eighteen patients received EMG biofeedback and seven patients received mirror biofeedback at home as a control group. The evaluation was performed after 1 year. The study showed that EMG biofeedback was more effective than mirror biofeedback (18).

*Dalla Toffola in 2005* conducted a study to compare two types of neuromuscular exercise therapy. Sixty-five patients with Bell’s palsy tested with electrodiagnosis were divided into two groups. Group 1 (28 patients) received stretch and active–assistive exercise kinesio therapy (KT). The average treatment session was 24 days. Group 2 (37 patients) received EMG biofeedback with an average treatment session of 17 days. Evaluation was performed using the House-Brackmann scale and grading of synkinesis. The study showed that EMG biofeedback was more effective and the degree of synkinesis was low in the biofeedback group (19).

## Discussion

All studies recommended exercise therapy, while some were based on exercise therapy. Methods were compared in all studies. Exercise therapy is the preferred treatment, although if it is used indiscriminately or instead of neuromuscular education, it causes mass movement (20). Incomplete recovery in FNP may not be due to muscle weakness, and if it is so, this weakness is in the secondary stage.

Unfortunately, facial muscles have few internal sensory receptors (20-21) and, unlike to other skeletal muscle, facial muscles are very small and delicate with minimal contractions and are high risk of contracture and changes in movement pattern. Uncontrolled synkinesis causes tension and changes in facial expression in the static and dynamic posture. Therefore, the main pathological problem in synkinesis concerns untimely charges of muscles; Furthermore, asymmetry in the face is not only due to muscle weakness but also concerns with in coordination between two sides of face. 

The results are mass movement, and in coordination between muscles. Therefore coordination and muscle timing should form the main components in treatment. For this reason some researchers suggest mime therapy or biofeedback therapy (EMG biofeedback or mirror biofeedback) for control, reducing synkinesis and symmetry in the face. In fact, neuromuscular reeducation has priority to exercise therapy and with repetition it cause strengthening. In other words, this form of neuromuscular re-education could be a reflection of normal or abnormal movement and allows the patient to represent continuous covert physiologic events about which he would normally be unaware. It is also possible to train inhibition of motor muscle behavior such as synkinesis.

## Conclusion

This study shows that patients with incomplete recovery of FNP require rehabilitation treatment. The best treatment protocol among rehabilitation program is biofeedback.
